# Converting Physical Function Testing to the Remote Setting: Adapting Our Research Protocol During COVID-19

**DOI:** 10.1093/geroni/igaa057.3431

**Published:** 2020-12-16

**Authors:** Kerri Winters-Stone, Colin Lipps, Carolyn Guidarelli, Pablo Herrera-Fuentes

**Affiliations:** 1 Oregon Health & Science University, Portland, Oregon, United States; 2 Oregon Health & Science University, portland, Oregon, United States; 3 Oregon Health & Science University, PORTLAND, Oregon, United States

## Abstract

Objective measurement of physical function can be a more sensitive predictor of future disability and changes over time than self-report measures. However, objective measures require in-person assessments which can limit their use in hard-to-reach populations. During the COVID-19 pandemic, laboratory assessment of physical functioning in our two large randomized controlled exercise trials in older adults with cancer was temporarily suspended. We adapted testing protocols for administering the short physical performance battery (PPB), including chair stand (CS; sec) and 4m usual walk (4MW; m/s) tests, and timed-up-and go (TUG; sec) tests of physical functioning for remote assessment by video conferencing technology. We report on interim assessments of inter and intra-rater reliability and validity against newly resumed in-person tests. Repeat assessments were conducted on the same participant 1-2 weeks apart, by the same assessor (intra-rater reliability) or by another assessor (inter-rater reliability) for remote visits and by the same assessor for in-person tests (validity). Pearson-product moment correlations are provided for PPB, CS, 4MW and TUG, in that order. Intra-rater reliability values are: 0.97, 0.88, 0.90, and 0.96; Inter-rater reliability values are: 0.79, 0.14, 0.40, and 0.99; Validity values (no TUG) are: 0.66, 0.81, 0.78. Reliability of conducting remote PPB by video is very high when using the same assessor, while TUG reliability is high within and between assessors. Remote PPB administration has a good correlation with in-person visits, but the two modes of delivery should not yet be used interchangeably in longitudinal studies until sources of error can be determined and minimized.

